# A Digital Peer Support Platform to Translate Web-Based Peer Support for Emerging Adult Mental Well-being: Protocol for a Randomized Controlled Trial

**DOI:** 10.2196/34602

**Published:** 2022-09-20

**Authors:** GeckHong Yeo, Weining Chang, Li Neng Lee, Matt Oon, Dean Ho

**Affiliations:** 1 N.1 Institute for Health National University of Singapore Singapore Singapore; 2 The Institute for Digital Medicine Yong Loo Lin School of Medicine National University of Singapore Singapore Singapore; 3 Office of Chief Psychologist Ministry of Social and Family Development Singapore Singapore; 4 Department of Psychology National University of Singapore Singapore Singapore; 5 Acceset Pte Ltd Singapore Singapore; 6 Department of Biomedical Engineering National University of Singapore Singapore Singapore

**Keywords:** mental health, digital health, peer support intervention, peer emotional disclosure, randomized controlled trial

## Abstract

**Background:**

Mental health issues among emerging adults (aged 19-25 years) on a global scale have underscored the need to address their widespread experiences of depression and anxiety. As a result of the COVID-19 pandemic, emerging studies are being directed toward the development and deployment of digital peer emotional disclosure and support for the psychological well-being of emerging adults. However, it is important to explore the implementation and clinical effectiveness, as well as associated mechanisms of change, for optimal approaches in conducting digital peer support interventions for emerging adults’ psychological well-being.

**Objective:**

We describe a randomized controlled trial to evaluate the implementation and clinical effectiveness of Acceset, a digital peer support intervention to address emerging adult mental well-being. The intervention has 2 components. First, the digital peer support training equips befrienders (ie, peers who provide support) to harness 4 components of psychological well-being—mattering, selfhood, compassion, and mindfulness—to provide effective peer support for seekers (ie, peers who seek support). Second, Acceset incorporates psychological well-being digital markers and harnesses community engagement to drive emotional disclosure among peers.

**Methods:**

A total of 100 participants (aged 19-25 years) from the National University of Singapore will be recruited and randomized into 2 arms. In arm 1 (n=50), the seekers will use Acceset with befrienders (n=30) as well as moderators (n=30) for 3 weeks. Arm 2 comprises a wait-listed control group (n=50). A questionnaire battery will be used to monitor seekers and befrienders at 4 time points. These include baseline (before the intervention), 3 weeks (end of the intervention), and 6 and 9 weeks (carryover effect measurement). Implementation outcomes of the intervention will involve evaluation of the training curriculum with respect to adoption and fidelity as well as user acceptability of the Acceset platform and its feasibility for broader deployment. Clinical outcomes will include mattering, selfhood, compassion, mindfulness, perceived social support, and psychological well-being scores.

**Results:**

This protocol received National University of Singapore Institutional Ethics Review Board approval in October 2021. Recruitment will commence in January 2022. We expect data collection and analyses to be completed in June 2022. Preliminary findings are expected to be published in December 2022. The Cohen *d* index will be used for effect size estimation with a .05 (95% reliability) significance level and 80% power.

**Conclusions:**

This protocol considers a novel digital peer support intervention—Acceset—that incorporates components and digital markers of emerging adult mental well-being. Through the validation of the Acceset intervention, this study defines the parameters and conditions for digital peer support interventions for emerging adults.

**Trial Registration:**

ClinicalTrials.gov NCT05083676; https://clinicaltrials.gov/ct2/show/NCT05083676

**International Registered Report Identifier (IRRID):**

PRR1-10.2196/34602

## Introduction

### Background and Rationale

Emerging adulthood is a development phase that spans the ages of 19 to 25 years in most high-income economies [1]. This developmental period is marked by unique challenges as emerging adults negotiate different features such as identity exploration and feelings of instability [1]. The uncertainty that accompanies the numerous changes and the substantial fluctuations in positive and negative emotional states during this developmental period result in widespread experiences of anxiety and depressed moods [1]. The global rise in mental health issues among emerging adults has fueled concerns regarding emotional distress, including depression, anxiety, and coping mechanisms [1]. This prepandemic emotional distress has been compounded by the outbreak of COVID-19, which has brought about prolonged isolation and reduced social connections [2-4]. With the unprecedented developmental implications associated with the pandemic most directly felt by college students negotiating emerging adulthood, there is a growing need to understand how to support the mental well-being of these young individuals [5,6].

Emerging studies are showing that digital peer support may effectively address the mental well-being of young people [7,8]. Digital communication has been prevalent among emerging adults even before the pandemic [9,10] and, with the onset of the pandemic, digital peer connections have become even more pervasive and important among college students as a coping mechanism to manage emotional experiences and distress [2]. Among college students, disclosure over digital communication channels is the norm for building relational closeness [11]. The collective experiences of managing intensifying concerns and intense negative feelings associated with the onset of the pandemic has driven college students’ social connections on the web [5,6], particularly the use of digital platforms for peer emotional sharing [2]. According to the social sharing of emotions framework, emotional disclosure on the web functions as a psychological mechanism for managing emotional lability and distress, which leads to emotional regulation and recovery [12,13]. However, it is important to increase our understanding of the ideal workflow for implementing digital peer emotional disclosure and support as an intervention [7,8], how to achieve relevant clinical outcomes, and the assessment of associated mechanisms of change. Therefore, it is essential to assess the efficacy of web-based peer emotional disclosure and support systems as well as the mechanisms of change.

### Importance of Digital Peer Emotional Disclosure and Support With the Onset of the COVID-19 Pandemic

According to the social sharing of emotions framework, emotional disclosure refers to the dyadic communication of significant and personal experiences with mild to strong positive and negative emotions with one’s close social network [12-14]. It focuses on emotionally laden experiences (eg, *I was exhilarated to receive a good grade on an exam* or *I was distressed being involved in a car accident*) that are distinguished from impersonal experiences (eg, *Football games can be exciting*) and those that are mundane and void of emotional content (eg, *I went to town after school*) [12-14]. For emerging adults, peers are the primary target audience for emotional disclosure, which serves as a stress coping mechanism that facilitates emotional regulation and recovery [12-14].

With the increased prevalence of digital peer communication among emerging adults, this form of communication has the potential to facilitate and sustain relational closeness and relationship quality with friends in a scalable manner [15,16]. A possible mechanism is the social sharing of emotions as emerging adults commonly disclose their emotions through texting [17]. The importance of digital peer emotional disclosure has been heightened with the onset of the COVID-19 pandemic. The social implications of the COVID-19 pandemic, which involve uncertainty, insecurity, and a reduced sense of agency and self-directedness, have had a substantial impact on emerging adults, especially college students [18,19]. Emerging adults reported increased loneliness as social and physical distancing were implemented, and staying connected with peers through digital communication may buffer the feelings of loneliness [5,6]. For college students, their peer emotional disclosure on the web has been pivotal in managing the collective experiences of intensified concerns and intense negative feelings [2].

Emotional lability in adolescence, which stems from extensive biological and social changes in relational dynamics with parents and peers, extends into emerging adulthood as a consequence of negotiating a different set of developmental features that are new and challenging [20,21]. Studies have indicated that emerging adulthood is characterized by heightened fluctuations in emotional positivity (ie, the degree of positive emotional experiences) and negativity (ie, the degree of negative emotional experiences), which are associated with a greater propensity for anxiety and depression than in other developmental stages [1]. These normative experiences have been compounded with the onset of the pandemic. Studies have documented systematic decreases in positivity and increases in negativity that were associated with COVID-19 among adolescents and college students [2-4]. These observations demonstrate the need to prioritize the development of mental health interventions for emerging adults.

Our review of the existing literature reveals a limited understanding of how dyadic emotional disclosure and support from friends can collectively function as a digital intervention in support of the mental well-being of emerging adults [8,22-24]. Before the pandemic, the administration of digital peer support to a college student population mediated the impact of various psychological and health outcomes [25,26]. With the onset of the global pandemic, the increase in social connections on the web underscores the potential effectiveness of digital peer emotional disclosure and support to positively affect the psychological well-being of college students [2,5,6]. Digital peer support platforms may address specific areas of well-being that include anxiety, depression, and suicidal ideation [24,27]. These findings have provided the impetus for researchers, education stakeholders, and health care professionals to understand the role of peer support on digital platforms in intervening in the psychological well-being of emerging adults, particularly college students. The subsequent initiation and completion of these studies are expected to yield insights into the implementation and feasibility outcomes, user acceptability of the interventions, and clinical effectiveness in addressing anxiety and depression among college students.

### This Study

#### Overview

Research evidence indicates that interventions that successfully affect the mental well-being of emerging adults include the following elements: (1) multiple components; (2) community engagement; and (3) consultation with emerging adults on the co-design of the intervention in an effort to potentially increase adherence and sustained engagement with the platform, a critical element of scalable and effective interventions [8,28,29]. To this end, we worked with Acceset (Multimedia Appendix 1), a social enterprise that provides digital text-based intervention to harness the therapeutic potential of peer disclosure and support on the web for emerging adult mental well-being. Through Acceset, users (seekers) can anonymously share their emotional experiences and receive support from their peers (befrienders), who receive training in digital peer support skills from clinical psychologists and certified counselors (moderators). Aligned with the characteristics of successful interventions, Acceset has been designed as a digital peer support intervention that is multifaceted. It entails a digital peer support training curriculum and a digital text-based intervention, uses a community-based approach, and involves emerging adults in the design and trial process. In this study, Acceset is a stand-alone product that is used as an intervention platform.

The digital peer support training being validated in this study may serve as a comprehensive strategy built on anonymous human interaction to address mental well-being in that it harnesses 4 components of emerging adult mental well-being. Emerging literature on COVID-19 underscores the role of these components in alleviating emerging adults’ psychological distress, including anxiety and depression [30-32]. These components include (1) enhancing one’s sense of mattering (the extent to which we are important to the surrounding world and people), (2) strengthening selfhood (one’s sense of identity and role), (3) exploring compassion (the degree of sensitivity to one’s and others’ pain and distress, with a desire to alleviate that pain and distress), and (4) cultivating mindfulness (paying attention to the present moment with intention and acceptance; refer to the Methods section for details). Our consultations and pilot trial of the Acceset intervention involving emerging adults from institutes of higher learning (IHLs) revealed findings that were consistent with the literature regarding how mattering, selfhood, compassion, and mindfulness can address the mental health challenges of college students [33], particularly during the global pandemic. The coding of the content from these emerging adults’ lived experiences of mental health conditions, particularly their anxiety and depressive symptoms, revealed these 4 components that helped them manage mental health challenges (Multimedia Appendix 2). These preliminary findings provide evidence to harness the interventional potential of these 4 components in Acceset digital peer support training to build befrienders’ capacity to deliver these ingredients to provide effective peer support to seekers.

The Acceset text-based intervention (ie, the platform) incorporates digital markers of psychological well-being—specifically, emotionality (ie, positivity and negativity), motivations, and functional adjustment (ie, internalizing and externalizing behaviors)—and hinges on the peer emotional disclosure process. These are markers of psychological well-being as emerging adults’ emotional positivity and negativity, innate psychological motivations, and functional adjustment are important precedents and indicators of their well-being [1,34,35]. During the course of the digital peer interaction, when users engage with the features on the Acceset platform, they provide information about these markers, which is subsequently correlated with their self-report measures of mental well-being. These markers can shift during the course of interaction for longitudinal assessment of user mental well-being status and can potentially also stratify users toward follow-up engagement with additional mental health professionals and resources. Furthermore, the Acceset digital peer emotional disclosure and support process leverages the Singapore ecosystem and community to potentially form a web-based safety net. Designed to reduce fear and stigma, seekers (those who seek help anonymously) find help through the exchange of anonymous e-letters by disclosing the issues that bother them. By tapping on common lived experiences, the community (befrienders and moderators) relates authentically to the seeker and affirms the seeker’s emotional experiences by giving dedicated attention to the issues with which they are confronted. As a key strategy for effective engagement with digital interventions is co-designing them [8,29,30], consultations with emerging adults aged 19 to 25 years from institutes of higher education in Singapore were conducted to curate these digital features.

#### Aims, Research Questions, and Hypotheses

The Acceset intervention addresses 3 gaps in the current research. First, our published work, undertaken to identify mobile health (mHealth) platforms across popular app stores and academic databases, found 302 anxiety and depression mHealth platforms that are currently available [36]. Of note, most mHealth platforms on the market are not designed for a specific age group and, even of those used academically, only 13% are designed specifically for emerging adults. Second, despite preliminary literature that highlights the importance of digital intervention, particularly involving peer support, on emerging adults’ psychological well-being, there is a continued need to achieve effective implementation strategies and clinical outcomes [24,27]. The increasing mental health issues that stem from prolonged isolation and reduced social connections associated with the outbreak of COVID-19 further the impetus among researchers, policy makers, and other stakeholders to design effective interventions that consider the need for users to access support remotely. To this end, a digital peer support intervention provides the desirable features of remote accessibility, which enables nonstrictly in-person mental health treatment options as well as remote monitoring access.

Third, notwithstanding the benefits of harnessing the components of mattering, selfhood, compassion, and mindfulness in promoting emerging adults’ mental well-being, the proposed validation of these combined components for emerging adults’ psychological well-being represents, to the best of our knowledge, a unique prospective study. A leap forward would be to test the feasibility of incorporating these components in an mHealth platform either as a stand-alone platform or as a combination intervention. Finally, in the absence of evidence demonstrating the mechanism of change relating digital peer support interventions to emerging adults’ psychological symptoms, it is not clear whether or how digital peer support intervenes in the development of psychological symptoms or how they unfold over time. This information could potentially provide actionable knowledge for timely and relevant digital peer support interventions. To this end, this study is based on the aims and hypotheses outlined in Textbox 1.

**Aims and hypotheses of this study.**

**Aims and hypotheses**

- Aim 1: the first aim is to evaluate the implementation effectiveness of digital peer support training between emerging adults providing support (befrienders) and emerging adults seeking help (seekers).
  Research question 1a: is Acceset digital peer support training an effective training curriculum in providing peer support?
  Hypothesis 1a: the digital peer support training is effective for befrienders in providing peer support, as demonstrated by the adoption of and fidelity to the training curriculum.
  Research question 1b: is the Acceset text-based intervention (ie, the platform) feasible and acceptable (ie, safe and timely) as an ongoing mechanism of support for seekers and befrienders?
  Hypothesis 1b: digital peer support is feasible and acceptable in offering support for emerging adults’ mental well-being, which is evidenced by 2 indicators:
    The engagement of the seeker-befriender-moderator interaction across the 4 time points—baseline (before the intervention), 3 weeks (conclusion of the intervention), and 6 and 9 weeks (carryover effect assessment)—that is indexed by the waiting period for seekers to receive a response. Delays are defined as a >48-hour waiting period and will be categorized based on the following reasons: (1) technical factors (eg, the befriender is not able to log into the platform to respond in time) and (2) human factors (eg, the befriender drops out from the study, and no replacement is found in time). An acceptable response time is within 48 hours, and this is emphasized during Acceset digital peer support training for befrienders [37].
    The use of technical features of the Acceset platform that is measured by the seeker dropout rate at each stage of engagement with the Acceset platform, the number of visits on the study registration website, the number of participants who registered with Acceset, the average number of letters exchanged, and the average number of emotion and functional adjustment stickers and motivation graphic interface formats used [37].
  Research question 1c: is the Acceset text-based intervention (ie, the platform) feasible and acceptable (ie, safe and timely) as an ongoing mechanism of support that identifies individuals who are at an unacceptably high risk of mental health conditions?
  Hypothesis 1c: digital peer support is feasible and acceptable in offering support for emerging adults’ mental well-being by identifying individuals who are at an unacceptably high risk of mental health conditions related to depression and suicidality, which is measured before and during engagement based on 3 means—specifically, the Acceset algorithm detection of the content of the letters, the moderators’ vetting of the content of seekers’ letters, and the seekers’ self-report responses to the 9-item Patient Health Questionnaire [38] (refer to the Implementation Outcomes section for details [37]).
- Aim 2: the second aim is to elucidate whether Acceset digital peer support harnesses the 4 components of emerging adult mental health—mattering, selfhood, compassion, and mindfulness.
  Research question 2a: does Acceset digital peer support enhance the 4 components among seekers and befrienders?
  Hypothesis 2a: Acceset digital peer support (training) increases the 4 components among befrienders (before and after training) and seekers (over the course of the study across 4 time points).
  Research question 2b: is Acceset digital peer support effective in improving emerging adult mental well-being?
  Hypothesis 2b: digital peer support leads to significantly better mental well-being of seekers compared with the control group. This effect is sustained beyond the period of the intervention.
- Aim 3: the third aim is to investigate the mechanism of change in emerging adult mental well-being involving digital peer support (delivered by the Acceset platform).
  Research question 3: what is the mechanism explaining the change in emerging adult mental well-being involving digital peer support?
  Hypothesis 3: the initial level and rate of change (ie, growth factors) of befrienders’ web-based support positively predict the growth factors of seekers’ psychological well-being in accordance with latent growth curve modeling.

## Methods

### Trial Design

This trial is registered with the US National Library of Medicine ClinicalTrials.gov (NCT05083676). We are initiating an interventional prospective study with a hybrid design that evaluates both the implementation of the Acceset intervention (eg, fidelity, adoption, and utility) and participant outcomes (eg, anxiety and depressive symptoms) via a randomized controlled trial (RCT). The intervention comprises 2 components—digital peer support training and a digital text-based intervention (ie, the platform)—involving 3 features: digital markers of psychological well-being, the peer emotional disclosure process, and community engagement.

### Participants and Study Setting

We plan to recruit 130 participants (100 seekers and 30 befrienders) at the National University of Singapore (NUS) to engage with the Acceset intervention in accordance with an Institutional Review Board–approved protocol. Participants will be recruited via the Research Participation program within the Department of Psychology at NUS, where students will be awarded credits as part of the introductory courses for psychology. During recruitment, inclusion and exclusion criteria screening will be conducted via self-reporting. Participants qualified for inclusion (seekers) will be randomized into 2 arms. The seekers (n=50), befrienders (n=30), and moderators (n=30) in arm 1 will use Acceset for 3 weeks. The control group (n=50) in arm 2 will be wait-listed for Acceset use. It should be noted that the wait-listing approach is a standardized approach for RCTs pertaining to digital mental health [37,39,40].

We will recruit age- and gender-matched individuals from NUS. The 4–time-point questionnaire battery of arm 1 will be used to compare the mental well-being and help-seeking behavior with those in arm 2 [37]. Moderators will be recruited from the Singapore Executive Counselling and Training Academy. If an unacceptably high risk of depression and suicidality is detected at any time point by the moderator, who is a certified counselor, they will reach out to the participant, who will be directed to different clinical mental health support providers, including counseling centers and hotlines, on the NUS campus. On the basis of power analysis and the finding that 20% to 40% of the emerging adults enrolled in an IHL typically experience distress, this sample will provide a sufficiently powered comparator group.

Upon recruitment, seekers will be able to register in two ways: (1) by engaging with a Telegram chat channel, an instant messaging app that allows users to send text messages, photos, videos, stickers, and files, which is a prevalent mode of communication among young people, and (2) through the Acceset website. The user will provide their email address, password, age, and gender. When using Telegram, the seekers will submit consent to the privacy policy and terms of use and acknowledgment that they fulfill the criteria for research participation (being aged 19-25 years and having no clinically diagnosed mental illnesses). During the intervention, seekers will be able to anonymously share their emotional experiences by writing e-letters. In response, they will receive support from a peer (befriender) who has received Acceset digital peer support training. When using the Acceset website, an automated bot will function as a courier to deliver the letter to the Acceset platform. The chatbot will also inform the user when they have a letter in the mailbox. The user will log in to retrieve the response letter from their mailbox.

The letter exchange will be monitored by moderators—certified counselors with the required background and assessment tools to identify seekers at risk of depression and suicidality. These moderators have received certified trainings that are accredited by the Australian, New Zealand, and Asian Creative Arts Therapies Association and Swinburne University of Technology in Professional Counselling; have a master’s degree in Social Science; and are certified by the Ministry of Education, Singapore, and the Executive Counselling and Training Academy to provide holistic counseling for students and supervision for counselors. Professionally, these moderators are experienced counselors at various IHLs; serve as vice-presidents of the Singapore Association for Counselling; and partner with schools and the Health Promotion Board, Singapore, to provide a range of counseling services to emerging and working adults.

The moderation protocol at the participant recruitment stage entails the screening of seekers using a validated psychological scale—the 9-item Patient Health Questionnaire (PHQ-9)—to exclude individuals at high risk of depression and suicidality. During the intervention phase, befrienders are also trained to alert moderators when seekers are at an unacceptably high risk of depression and suicidality, and the moderator vets all the letter exchanges between the seekers and befrienders for content of high negativity and suicidal thoughts and expressions. This moderation protocol aims to identify seekers who are at an unacceptably high risk of depression and suicidality and refer them to an appropriate mental health care provider, including counseling centers and hotlines, on the NUS campus.

Each befriender will provide support to 3 seekers, receive notification from the Acceset platform on incoming letters, and work in partnership with the moderators to address the seekers’ issues during the 3 weeks of letter exchange. Befrienders have the choice to discontinue the letter exchange with seekers if they feel discomfort having to address issues that they find challenging by informing trained moderators. Given the sensitive nature of the letter content, there may be issues that befrienders will be exposed to that they are not fully comfortable with and may cause distress. Therefore, it is a deliberate design to ensure that the befrienders are not left on their own in handling cases and that they work in partnership with the 2 trained and certified moderators to address the seekers’ issues. The first point of contact for the befriender will be the 2 trained and certified moderators. In such cases, the befriender’s workload decreases to 2 seekers.

The system will inform the seeker and obtain their consent to continue the conversation with a new befriender or end the letter thread. If the seeker chooses to continue, the system will notify all befrienders of the availability of an ongoing case, and interested befrienders can take it up. In addition, other trained befrienders (whom Acceset has trained beyond the recruited 30 befrienders for this study) will be activated to take up the case if no existing befrienders take it up. Similarly, when befrienders drop out of the study during the 3 weeks of letter exchanges, they will be replaced with other trained befrienders. Befrienders’, seekers’, and moderators’ use of the Acceset platform is to remain completely anonymous such that the 3 parties do not have direct contact with one another, and seekers do not have discretion to switch befrienders on their own. In the event of disagreement between seekers and befrienders (eg, seekers are concerned with how their issues are not adequately addressed during the letter exchange), they will be able to notify the study team. The system will inform the seeker and obtain their consent to continue the conversation with a new befriender or end the letter thread. If the seeker chooses to continue, the system will notify all befrienders of the availability of an ongoing case, and interested befrienders can take it up. However, it should be noted that such situations will be rare given that moderators will vet all letter exchanges and work in partnership with befrienders to ensure that their responses are satisfactory. If seekers drop out of the study, their befrienders will continue with the reduced workload.

**Figure 1:**
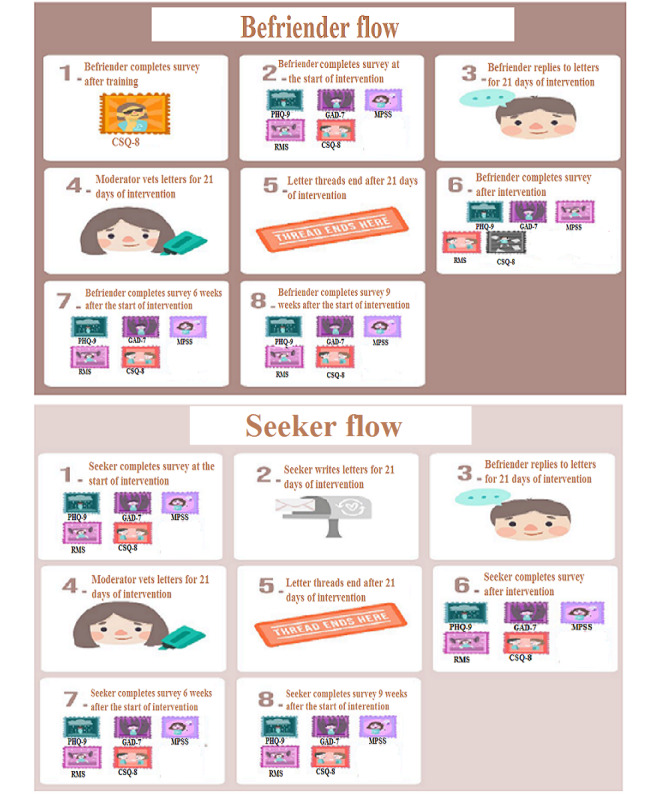
Diagrammatic flow in terms of sequence of events on the Acceset digital text-based intervention (ie, the platform). CSQ-8: Client Satisfaction Questionnaire-8; GAD-7: Generalized Anxiety Disorder-7; MPSS: Multidimensional Scale of Perceived Social Support; PHQ-9: 9-item Patient Health Questionnaire; RMS: Rosenberg Mattering Scale.

Befriender engagement will be monitored, and they will receive a reminder email after 48 hours if there is no reply to the seeker. A questionnaire battery over 4 time points will be used to monitor befrienders and seekers (Multimedia Appendix 3). These time points will include baseline (before the intervention), 3 weeks (end of the intervention), and 6 and 9 weeks (carryover effect measurement; Figure 1) [37]. Signals for closure will be done through an email reminder sent 7 days before the conversation ends, and another email will be sent to inform seekers and befrienders when the thread officially closes after 21 days. Where there are issues that reflect high negativity and suicidal thoughts and expressions at any point in the letter thread, we will immediately link the seekers to the appropriate professional counseling resource.

### Ethics Approval

This study will be performed in line with the principles of the Declaration of Helsinki. Approval was granted by the Ethics Committee of the NUS (protocol S-20-144). Informed consent will be obtained from all individual participants included in the study.

### Eligibility Criteria

The seeker inclusion criteria are as follows: emerging adults aged 19 to 25 years who are distressed but healthy—specifically, these individuals should exhibit some symptoms but have not been formally diagnosed with depression, anxiety, or a mental health disorder. The exclusion criteria (seekers) will include emerging adults who, during screening, are deemed to be at a high risk of suicide using a validated and standardized psychological scale (PHQ-9) [37,38]. On the basis of empirical studies, the proposed cutoff is PHQ-9 >9, which has a sensitivity of 88% and specificity of 88% for major depression [37]. Above a score of 9, seekers will not be recruited for the study, and they will be referred or directed to different clinical mental health support providers, including counseling centers and hotlines, on the NUS campus.

The rationale for including individuals who do not meet the criteria for depression is that the Acceset intervention focuses on befrienders as peer supporters to seekers, and these befrienders are not certified and accredited counselors, unlike moderators. Importantly, it is not the responsibility of the befriender to serve as an advisor, solve the seekers’ problems, or provide diagnoses of potential mental health issues. Inclusion of individuals who meet the criteria for depression would require support from professional counselors who have formal trainings and certifications. However, Acceset as an intervention platform is designed primarily for a healthy population of young people without formal diagnoses of mental health conditions to provide peer support.

The inclusion eligibility criteria also apply to befrienders, with an additional criterion of being aged ≥21 years. Seeker exclusion criteria apply to both befrienders and moderators. Moderator inclusion criteria are as follows: adults aged ≥21 years with certified trainings and accreditations (ie, at least a master’s degree in social sciences or Counseling that is recognized by the Ministry of Education, Singapore, and the Executive Counselling and Training Academy) and professional experiences in providing counseling services to emerging adults. The exclusion criteria apply to both seekers and befrienders.

### The Intervention

The Acceset intervention for this preliminary study comprises (1) training for befrienders to apply mattering, selfhood, compassion, and mindfulness to provide effective digital peer support (refer to the digital peer support training curriculum in Multimedia Appendix 4) and (2) a text-based intervention with Acceset incorporating digital markers that assess the following outputs: psychological well-being and the degree of community engagement as well as peer emotional disclosure [37].

### Digital Peer Support Training

The core objective of Acceset is to enable peer befrienders to effectively support peer seekers. Befrienders will receive 4 hours of web-based training conducted by a licensed clinical or educational psychologist. This training consists of 1 hour of instructional content that enhances the befriender’s knowledge of mattering, selfhood, compassion, and mindfulness and 2 hours of workshop training on the practical skills of applying these components to provide effective peer support.

Selfhood refers to self-knowledge (an awareness of one’s strengths and unique qualities) [41]. The second type of selfhood is the interpersonal self (how the self evolves based on our interactions and relationships with others). The interpersonal self creates and sustains relationships and fulfills important roles to keep a favored position in the social system. The third type of selfhood refers to the self as an agent (having agency, control, and persistence in achieving a goal despite failure, frustration, and discouragement). Mattering, which refers to the extent to which we are important to the world and people around us [42], consists of three components: (1) importance (the interest and concern others bestow on us), (2) attention (from others; the extent to which people are aware of our presence and unique qualities), and (3) reliance (the degree to which others turn to us and make us feel that we are needed).

Compassion comprises 3 theoretical orientations—having compassion for others, receiving compassion from others, and having self-compassion—in reducing psychological distress and symptomologies [43], and there are 3 stages of developing compassion based on the well-established Compassion Cultivation Training with evidence from accumulating RCTs [44]. In the first stage, individuals become aware of their distress and develop self-compassion; in the second stage, they develop affective concern; and, in the third stage, they gain personal insights into the wish and readiness to relieve one’s distress [44]. Mindfulness will be regarded as a flexible cognitive state in which individuals are actively present and notice novel aspects in both the environment and one’s perspectives [45].

The proficiency of befrienders in applying these digital support skills will be evaluated through simulation and homework activities in which they will apply these skills to simulated letters that are adapted from actual cases of peer seeker emotional disclosure on the Acceset platform. All assignments will be reviewed and given feedback on. At the end of the training, befrienders are expected to apply the 4 components to their assigned peers’ disclosed emotional experiences. All participants who complete the training will receive a training certificate and will be invited to take on live cases and be part of the study.

### Acceset Text-Based Intervention (the Platform)

Acceset aims to serve as an intervention to strengthen emerging adult mental well-being via 3 means—assessing their psychological well-being with 3 digital features: emotionality, motivations, and functional adjustment. These features are intended to support users with a peer emotional disclosure process while also engaging an interconnected and accessible Singapore ecosystem and community as the basis of the support system.

#### Digital Features

Participants’ emotionality, as reflecting their mental well-being, is assessed by their use of emotion stamps—a digital feature of the Acceset platform to measure negativity and positivity that is based on the Positive and Negative Affect Scale [46]. A total of 9 motivation graphic interface formats (GIFs)—dynamic images with corresponding captions—function to express a seeker’s motivation to engage with the Acceset platform. There are 3 innate psychological motivations [34]. These include competence (to exert control, cope with specific problems, and make changes to one’s behavior and environment), autonomy (to act from choice), and relatedness (the need to belong and connect with others). Acceset also has a set of functional adjustment stickers as indicators of internalized and externalized problems. Internalized problems refer to those directed inward—toward the self—and often manifest as “tension, unease, and distress” [47]. When these internalized symptoms compound, individuals may experience depression, anxiety, social isolation, and other challenges. On the contrary, externalizing problems are directed externally [48]. Individuals often disregard social norms and engage in conflicts and behaviors that cause discomfort to other people. When these externalizing problems are grouped together, they can potentially manifest as aggression, rule breaking, delinquency, and other behaviors.

#### Peer Emotional Disclosure

The Acceset text-based disclosure process collectively leverages technology and community engagement to form a web-based safety net for the development of a platform function that drives sustainable user engagement. This process begins when seekers engage with the Acceset platform to seek support with managing their emotional experiences. Before writing their first letter, seekers will select the social contexts of the issue. These may include friends, finances, family, work, and school. On the basis of the disclosed emotional experiences, the information presented will pertain to where the issues arise, with whom the issues occur, and which issues adversely affect mental well-being. During the course of the letter exchange, seekers will express their negativity and positivity through the emotion stamps, indicate their psychological needs using the motivation GIFs, and describe the effects of the emotional experiences through the function adjustment stamps.

The seeker-befriender interaction dynamic is facilitated digitally, with no in-person visits or interactions. Before and during the letter-writing process, a pop-up message reminds both befrienders and seekers that the use of the Acceset platform is to remain completely anonymous and that personal information may not be revealed in any way. In matching befrienders and seekers, 3 factors are considered: the timing when seekers reach out, the availability of befrienders, and befrienders’ knowledge of the nature of the seekers’ emotional experiences. Some befrienders may have more in-depth knowledge of certain issues based on their lived experiences and will prefer to support seekers on those issues. Thus, when the seekers write a letter, the social context of the letter is made known to the befriender, who will then select their preferred seeker based on their ability to relate and respond. The approach of matching befrienders and seekers is intended to facilitate effective peer support intervention [29]. Through the digital peer support training and the guidance of the moderators—certified counselors who will vet the e-letter drafts from the befrienders—the befriender’s role is to provide support by enhancing a sense of mattering, strengthening selfhood, exploring compassion, and cultivating mindfulness among seekers. Importantly, it is not the responsibility of the befriender to serve as an advisor, solve the seekers’ problems, or provide diagnoses of potential mental health issues.

#### Community Engagement

**Figure 2:**
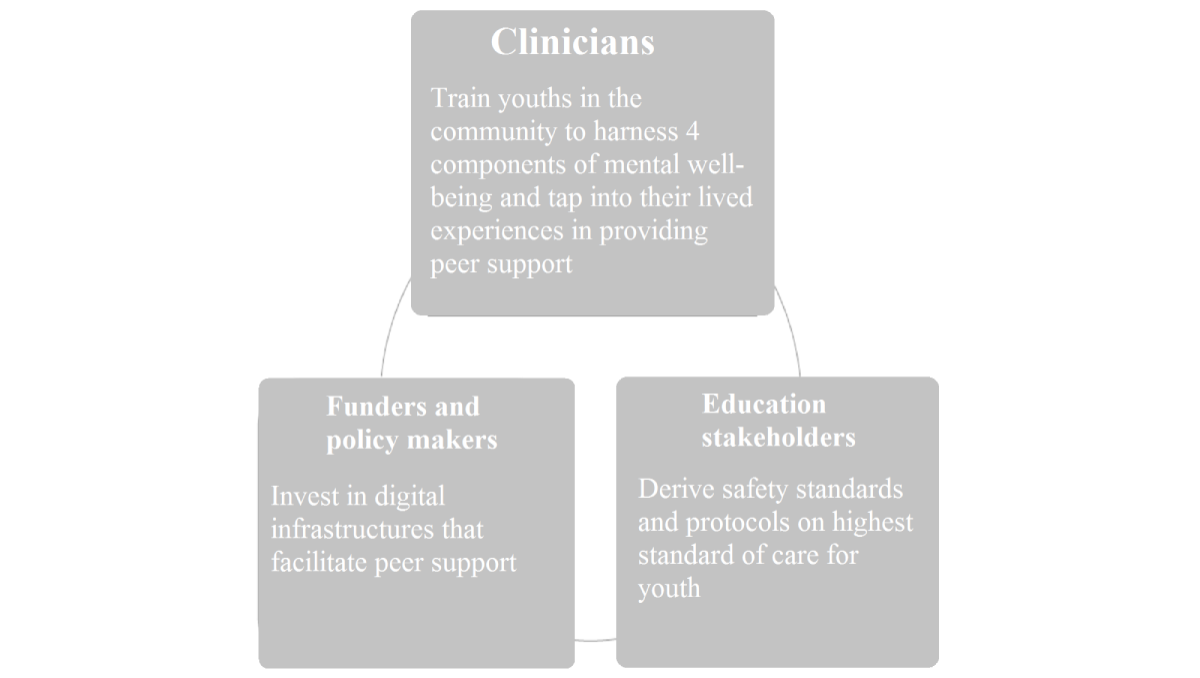
The Acceset intervention draws on digital peer support within the Singapore ecosystem and community.

The essence of the Acceset intervention draws on emerging adult support within the Singapore ecosystem and community by tapping into the shared experiences among emerging adults and training them to harness the 4 components of psychological well-being to bolster each other’s mental well-being (Figure 2). Seekers can further the community efforts by joining as befrienders and being trained to provide web-based peer support. By engaging clinicians, psychologists, and counselors, the Acceset intervention emphasizes the availability of peer support that leverages the lived experiences of the community. The building of the seeker-befriender-moderator relationship also aims to deepen the authenticity of peer support rather than being a “transaction” for referral, as evidenced by the effective implementation of the intervention when seekers’ and befrienders’ satisfaction with the support system and sense of perceived social support relate positively to their psychological well-being.

In addition to a community-based structure for facilitating digital peer support, Acceset engages education stakeholders at various IHLs in the advice of safety standards and protocols for effective and compliant digital peer support that is in accordance with the ethics of members of the helping profession. Education stakeholders play an important role in normalizing a new service delivery model through various formal and informal outreach channels. This could entail mass mailing of a new service and organization of an information session where students can learn more about the new service and how participating in it can be beneficial for their mental well-being. Attending college can be a particularly stressful time, especially in Asian societies with a high cultural value ascribed to educational achievement and a high level of competition [49,50]. The experience of heightened psychological distress is a salient problem, with an average of 20% to 40% of students reporting distressed symptoms [50]. This finding, coupled with the increasing number of emerging adults enrolled in 2- or 4-year colleges (40% in 2013 to 70% in 2018) [51], provides the impetus for leveraging a community approach in the development of digital peer support that intervenes in emerging adults’ psychological well-being.

### Implementation and Clinical Study Outcomes

All implementation and clinical outcomes will be measured via the engagement with Acceset over 4 time points using the aforementioned questionnaire battery [37]. In this section, we delineate the outcome measures that correspond to each hypothesis examined in this study.

#### Implementation Outcomes

For research question 1a and hypothesis 1a, we will determine whether Acceset digital peer support training is an effective curriculum for befrienders in providing effective peer support. We will assess the adoption of and fidelity to the training curriculum through the letter exchanges between seekers and befrienders. The text of the letter exchanges will be evaluated in terms of the extent to which befrienders display fidelity in applying the 4 components of psychological well-being in response to seekers’ disclosure. All correspondence will be anonymized before analysis, and the seekers’ letters will only be used to provide content for the befrienders’ responses. With respect to research question 1b, hypothesis 1b, research question 1c, and hypothesis 1c, we will evaluate the feasibility and acceptability of sustaining access to web-based peer support in terms of (1) initial and sustained engagement of seekers, befrienders, and moderators (ie, certified counselors); (2) the use of technical features of the Acceset platform; and (3) the identification of participants with high risk of mental health conditions relating to depression and suicidality.

This study will assess the waiting period for a seeker to receive a response. Delays (defined as a >48-hour waiting period) will be categorized based on the following reasons: (1) technical factors (eg, the befriender is not able to log into the platform to respond in time) and (2) human factors (eg, the befriender drops out from the study, and no replacement is found in time). An acceptable response time is within 48 hours, and this is emphasized during Acceset digital peer support training for befrienders. The seeker dropout rate at each stage of engagement with the Acceset platform will be assessed based on (1) the number of visits to the study registration website, (2) the number of participants who registered with Acceset, (3) the average number of letters exchanged, and (4) the average number of emotion and functional adjustment stickers and motivation GIFs used.

With regard to user referral to appropriate mental health support providers, the seeker-befriender-moderator interaction will identify participants at an unacceptably high risk of depression and suicidality before and during engagement based on 3 means. First, the Acceset algorithm will scan the content of the letter exchanges for high negativity and suicidal thoughts and expressions. Second, moderators with the required background and assessment tools will identify seekers at risk by vetting all the letter exchanges for high negativity and suicidal thoughts and expressions. Third, seekers will self-report their responses to the PHQ-9, and those that meet the clinical cutoff scores (PHQ-9 >9) will be excluded at the start of the study. During the course of the study across the 4 time points, seekers with an unacceptably high risk of depression and suicidality (PHQ-9 >9) will be referred to an appropriate mental health care provider, including counseling centers and hotlines, on the NUS campus. Seekers’ self-reported responses to the PHQ-9 serve as the primary risk assessment to make referrals to appropriate mental health support providers.

At recruitment, we will assess the percentage of participants identified as being at an unacceptably high risk of depression and suicidality meeting the clinical cutoff of PHQ-9 >9 that has a sensitivity of 88% and specificity of 88% for major depression [38] and exclude them from the study. During the course of the study, we will ascertain the percentage of events when a befriender correctly alerts the moderator that the seeker is at an unacceptably high risk of depression and suicidality as confirmed by the moderator, who will vet every letter exchange. During the course of the study and for every letter exchange, we will tabulate the percentage of events when a moderator correctly identifies a seeker with an unacceptably high risk of depression and suicidality. Both the befrienders and moderators will use the PHQ-9 as a systematized coding structure to code seekers’ letter content to ensure generalizability and validity in risk assessment across both coders—befrienders and moderators. Finally, we will tabulate the percentage of events when a seeker who is at an unacceptably high risk of depression and suicidality (both during the recruitment process and during engagement with the platform) receives directions to an appropriate mental health care provider, including counseling centers and hotlines, on the NUS campus.

The study team will alert the counseling center on the NUS campus about the referrals of at-risk seekers to ensure that adequate and appropriate support is provided to them. In addition, at weeks 6 and 9, there will be a follow-up on all seekers (to measure carryover effects), which helps ensure the psychological safety of the seekers. At the start of the study, all seekers are provided with the participant informed consent and information sheet, which states that they will be referred to and their information shared with the NUS counseling center if their risk assessment across the 4 time points indicates an unacceptably high risk of depression and suicidality (PHQ-9 >9).

#### Clinical Outcomes

To address research question 2a and hypothesis 2a, we will examine whether the Acceset training curriculum can enhance the 4 components of psychological well-being among befrienders and seekers. We will compare the change in mattering, selfhood, compassion, and mindfulness scores of befrienders between baseline and following Acceset training (ie, before vs after training) and those of seekers over the course of the study. As for research question 2b and hypothesis 2b, we hypothesized that engagement with Acceset digital peer support will lead to improved mental well-being of the seekers compared with the control group and that this effect may be sustained beyond the period of the intervention. To assess these effects, we will evaluate the change in mental well-being of the participants in both study groups (intervention and control) after 3, 6, and 9 weeks from baseline using self-report questionnaires. Seekers will provide responses pertaining to help-seeking behavior beyond the Acceset platform at weeks 6 and 9 using the perceived social support measure in the questionnaire battery. We will assess the correlations of the 3 digital features—comprising emotion and functional adjustment stickers and motivation GIFs, which reflect seekers’ mental well-being during engagement with the platform—with their well-being scores from the questionnaires.

The scale or set of scales used to assess the clinical outcomes, including the 4 components of psychological well-being underpinning the Acceset training curriculum, measures of seekers’ well-being, and perceived social support, are outlined in the following paragraph.

“Mattering” will be assessed via the Rosenberg Mattering Scale [52]. *Selfhood* comprises self-knowledge, interpersonal self, and self-agency and will be assessed via the following respective scales: the Rosenberg Global Self-Esteem Scale [53], the Self-Consciousness Scale [54], and the General Self-Efficacy and Social Self-Efficacy Scales [55]. *Compassion* will be assessed as operationalized in the Compassion Cultivation Training [56] comprising three stages: (1) an awareness of distress, (2) affective concern, and (3) a responsiveness or readiness to help relieve that distress (motivational). *Mindfulness* will be regarded as a flexible cognitive state in which individuals are actively present and notice novel aspects in both the environment and one’s perspectives [45]. We will evaluate the participants’ responses based on the degree to which they harness the approaches pertaining to the enhancement of mindful self-acceptance: (1) identifying novel aspects of the situation or perspective; (2) demonstrating “work in progress” by using possibility words such as “could be” and offering other interpretations of the situation their peers have shared; (3) highlighting puzzles and paradoxes in the peer-disclosed emotional experiences (eg, how their peers may feel victimized yet are responsible for being in that situation), which builds tolerance for ambiguity and decreases the experience of psychological symptoms; (4) noticing humorous aspects of the situation; (5) perceiving the situation from multiple perspectives; (6) considering alternative (useful) aspects of a problematic context or the silver lining; (7) emphasizing a mental file of positive memories; and (8) encouraging peers in mindfulness journaling. *Anxiety* will be measured using the Generalized Anxiety Disorder Questionnaire [57]. *Depression* will be measured using the PHQ-9 [38]. *Perceived social support* will be assessed using the Multidimensional Scale of Perceived Social Support [58].

#### Mechanism of Action

**Figure 3:**
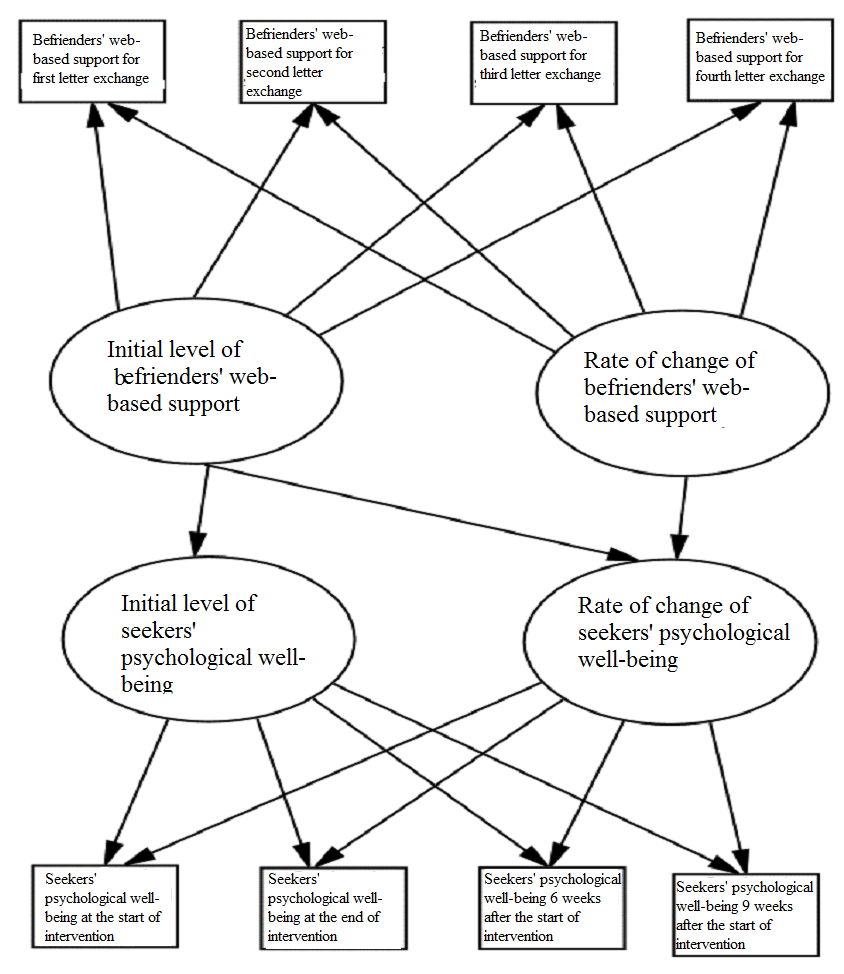
A mechanism of change linking befrienders’ web-based support and seekers’ psychological well-being.

For research question 3 and hypothesis 3, we will elucidate the mechanism of change that links digital peer support intervention to emerging adult mental well-being (Figure 3). Specifically, we will assess whether and how the level at the start of the study and the rates of change from baseline to weeks 3, 6, and 9 in befrienders’ support relate to seekers’ mental well-being.

### Data Collection, Management, and Analyses

Acceset will serve as the data collection platform. Participants (both seekers and befrienders) will initially enter their details on the sign-up page, which includes role preference (seeker, befriender, or either), email, date of birth, gender, nationality, ethnicity, monthly household income, and password. The user will be required to answer the PHQ-9 to determine their eligibility. An email will also be sent to the users’ registered email address for verification. Once eligible individuals are selected and assigned their respective roles, they will be given a unique nonidentifying ID to participate in the study. Participants’ self-reported questionnaire responses at each time point and the digital letter exchange content will be securely stored and tied to their specific IDs. The data collected will be stored on the cloud servers of the leading author’s affiliated institution. Once the research concludes, the information will be downloaded to a Microsoft Excel sheet, and all personal information will be deidentified for data analyses.

A power analysis assessed the adequate sample size needed for the RCT to have sufficient power to detect valid effects. The intervention (n=50) and control (n=50) arms were derived from multiple dependent variables assessing implementation testing (ie, feasibility and utility) and clinical outcomes. To address any potential challenges with missing data, the Little Missing Completely at Random Test will address potential missing data via full information maximum likelihood imputation [37,59]. Maximum likelihood estimation is a method that ascertains the parameter values of a model using the mean and variance and maximizes the chance that the values generated are closest to those observed.

The following approaches will be used for data analysis and interpretation. Independent-sample 2-tailed *t* tests will compare the intervention and control groups. Bonferroni post hoc tests will control for multiple comparisons. For both the intervention and control groups, additional independent *t* tests will compare seekers who provided data across all 4 time points with those who dropped out from the study for different reasons to ascertain if the sample with data across all 4 time points and the sample with fewer data points bias our results. For instance, seekers may drop out when risk assessments identify them as having a high risk of depression and suicidality during the course of the study. There will also be instances when befrienders discontinue the letter writing because of the sensitive nature of the letter content that may cause distress and befrienders not being fully comfortable, and the system will inform the seekers and obtain their consent to end the letter thread instead of continuing the conversation with a new befriender. Second, each participant’s emotional experiences disclosed on Acceset will be extracted based on the definitions of the 4 components of psychological well-being. Latent Dirichlet allocation analyses in R (R Foundation for Statistical Computing) and content analysis will be used [37,60,61].

Latent growth curve modeling (LCM) will investigate the mechanism of change involving the trajectories of peer support in predicting the change in psychological well-being [59]. LCM delineates whether and how the exposure to the intervention changes over time with psychological outcomes by estimating and comparing different baseline latent growth models, including linear, quadratic, and nonlinear curve fitting (ie, optimal fit). In other words, LCM elucidates whether the trend in befrienders’ peer support predicts the trend in seekers’ psychological well-being (Figure 3). Current research assessing peer support intervention for the mental health of young people does not consider the mechanism of change. By establishing a change model, this protocol provides a basis for building the therapeutic potential of digital peer support that harnesses the components of emerging adult mental well-being to reduce psychological symptoms. Specifically, LCM elucidates whether and how the intervention affects the development of psychological symptoms and how they unfold over time among emerging adults. This finding provides actionable knowledge for timely and relevant digital peer support.

## Results

This protocol received approval from the Institutional Ethics Review Board of NUS in October 2021. Recruitment will commence in January 2022. Data collection began in March 2022 and was completed in June 2022. Data analysis started in July 2022 and is currently in progress. We aim to report the preliminary study outcomes in December 2022. The effect size will be analyzed using the Cohen *d* index with a significance level of .05 (95% reliability) and 80% power statistic [37]. A total of 100 seekers, 35 befrienders, and 2 moderators were recruited in January 2022.

## Discussion

### Principal Findings

This protocol delineates the design of an RCT to assess the implementation and clinical effectiveness of Acceset. To our knowledge, the proposed intervention is the first to incorporate and validate digital markers of psychological well-being, harness components of emerging adult mental well-being, and elucidate a mechanism of change involving digital peer support in mitigating emerging adults’ psychological symptomologies. We hypothesized the following: (1) the digital peer support training will be effective for befrienders in providing peer support, as demonstrated by the adoption of and fidelity to the training curriculum with significantly higher scores after training than before training on the 4 components of well-being (selfhood, mattering, compassion, and mindfulness); (2) the digital peer support intervention will be feasible and acceptable in offering support for emerging adults’ mental well-being through the engagement of seeker-befriender-moderator interaction over the Acceset platform throughout the course of the study (baseline [before the intervention], 3 weeks [the end of the intervention], and 6 and 9 weeks [to measure carryover effects]) and the use of the platform’s technical features; (3) the digital peer support intervention will be feasible and acceptable in offering support for emerging adults’ mental well-being by identifying individuals who are at an unacceptably high risk of mental health conditions related to depression and suicidality based on the Acceset algorithm, moderators’ vetting of the letter exchanges, and seekers’ self-report responses to the PHQ-9; (4) the digital peer support intervention will lead to significantly better mental well-being of seekers compared with the control group, with the effect sustained beyond the period of the intervention; and (5) the mechanism of change, as indexed by LCM, will reveal that the initial level and rate of change (ie, growth factors) of befrienders’ web-based support positively predict the growth factors of seekers’ psychological well-being.

If successful, this novel mHealth intervention will provide the parameters and conditions required to validate the effectiveness of digital peer support interventions in real-world settings for emerging adult mental well-being. This intervention may, in turn, represent a scalable, sustainable, and low-cost prevention strategy that has considerable potential to support positive psychological well-being among young people, especially in coping with the life-course implications of the global pandemic.

### Strengths, Limitations, and Conclusions

The key strengths of the proposed intervention are the scalability and sustainability of both the Acceset digital peer support training and text-based intervention (ie, the platform). Existing evidence suggests that, for the scalability of web-based nonprofessional peer support training to attain the desired reach, training should incur minimal cost and be available to individuals across geographical locations and from diverse backgrounds and abilities [22,62]. Consistent with these findings, the digital peer support training is designed to maximize reach and is widely accessible at scale. A benefit of using a web-based format for the 3 hours of training is its link to existing IHL curricula that are comprised of 1 hour of lecture and 2 hours of tutorial discussion. This format may enable IHLs to serve as key delivery partners in rolling out the training program on the web for diverse student populations and across physical locales. Importantly, existing findings suggest that digital mental health interventions that harness peer support and adopt dual community engagement approaches (active and consultative) drive optimal user engagement [28]. The Acceset intervention uses the active method of community engagement to provide web-based safety support for young people. This form of peer support is affordable and readily available as it draws on the common lived experiences of the community, with peers functioning as befrienders and moderators who are licensed mental health professionals or counselors to provide their fellow peer seekers with emotional support. By using the community consultative method, college students aged 19 to 25 years from IHLs in Singapore were consulted in co-designing the Acceset platform.

Results from a systematic review and meta-analysis of RCTs on the sustainable effects of mental health interventions for students from IHLs emphasize the significance of a multisystemic approach [63-65]. This approach entails the contributions from the individual, community, and societal levels to maximize the effectiveness and sustainability of psychological interventions for mental health, especially for emerging adults [63-65]. The web-based peer emotional disclosure and support system of the Acceset intervention is envisioned to be self-sustainable as the seeker-befriender-moderator dynamic is characterized by individual contributions, with the opportunity for seekers to join the peer support network as befrienders to expand the web-based network. In addition, the Acceset intervention taps into the community and society levels by leveraging technology and engaging clinicians, psychologists, and counselors in the community to further the sustainability of digital peer support. At the macrosystemic level, Acceset serves as a platform for community-driven cocreation, validation, and potential deployment. Collaborations with policy makers and IHLs in designing safety standards and protocols for emerging adults engaged in digital peer support may bolster platform sustainability by normalizing a new service delivery model.

A possible limitation of this study is the use of self-reported measures for the clinical outcomes of this trial, which could be subject to under- or overestimation of psychological well-being scores. This approach is necessary because of confidentiality and feasibility considerations, especially with the anonymity of the digital letter exchange on the Acceset platform, and is consistent with the standard protocol of school-based trials for peer-led mHealth interventions for young people [62]. To reduce self-report bias, whenever seekers and befrienders log in to the Acceset text-based platform both before and for the duration of the study, pop-up messages function to remind them of strict anonymity, with no in-person interactions or sharing of personal information that will reveal their identities.

The results of this study on validating the digital markers of psychological well-being on the Acceset platform could potentially be used to triangulate evidence with self-report measures and biomarkers of psychological well-being. Future research may continue to accumulate evidence on these digital markers of psychological well-being and incorporate biomarkers such as cortisol levels and blood pressure to provide a different method of data collection to enhance the validity and reliability of clinical outcomes on the mental well-being of young people. The evidence that is built on a single-site superiority trial outlined in this protocol limits the external validity of digital peer support interventions in real-world settings [28]. Future research should consider properly powered and rigorous studies using multiple trials of strategies and multiple sites to build the evidence on the effects of digital interventions addressing emerging adult mental health. Notwithstanding these limitations, this study’s development and validation of a novel digital innovation for emerging adult mental well-being may provide important contributions to the field of mental health [8,28].

## Appendix

Multimedia Appendix 1 Information on Acceset—a social enterprise that provides digital text-based peer support intervention for emerging adult mental well-being.

Multimedia Appendix 2 Components extracted from consultations with emerging adults about their lived experiences of mental well-being.

Multimedia Appendix 3 Questionnaire battery to assess the variables in the study.

Multimedia Appendix 4 Digital peer support training curriculum.

## Abbreviations

GIF: graphic interface format
IHL: institute of higher learning
LCM: latent growth curve modeling
mHealth: mobile health
NUS: National University of Singapore
PHQ-9: 9-item Patient Health Questionnaire
RCT: randomized controlled trial
