# Supplementary material for: A Digital Peer Support Platform to Translate Web-Based Peer Support for Emerging Adult Mental Well-being: Protocol for a Randomized Controlled Trial
Source: JMIR Res Protoc. 2022 Sep 20;11(9):e34602. doi: 10.2196/34602 (PMC9533208; doi:10.2196/34602)
Supplement: Multimedia Appendix 1 [file resprot_v11i9e34602_app1.pdf]

acceset

accept care & reset lives

Matt Oon

Founder & CEO

www.acceset.com

matt@acceset.com

Anonymous Peer Support Platform

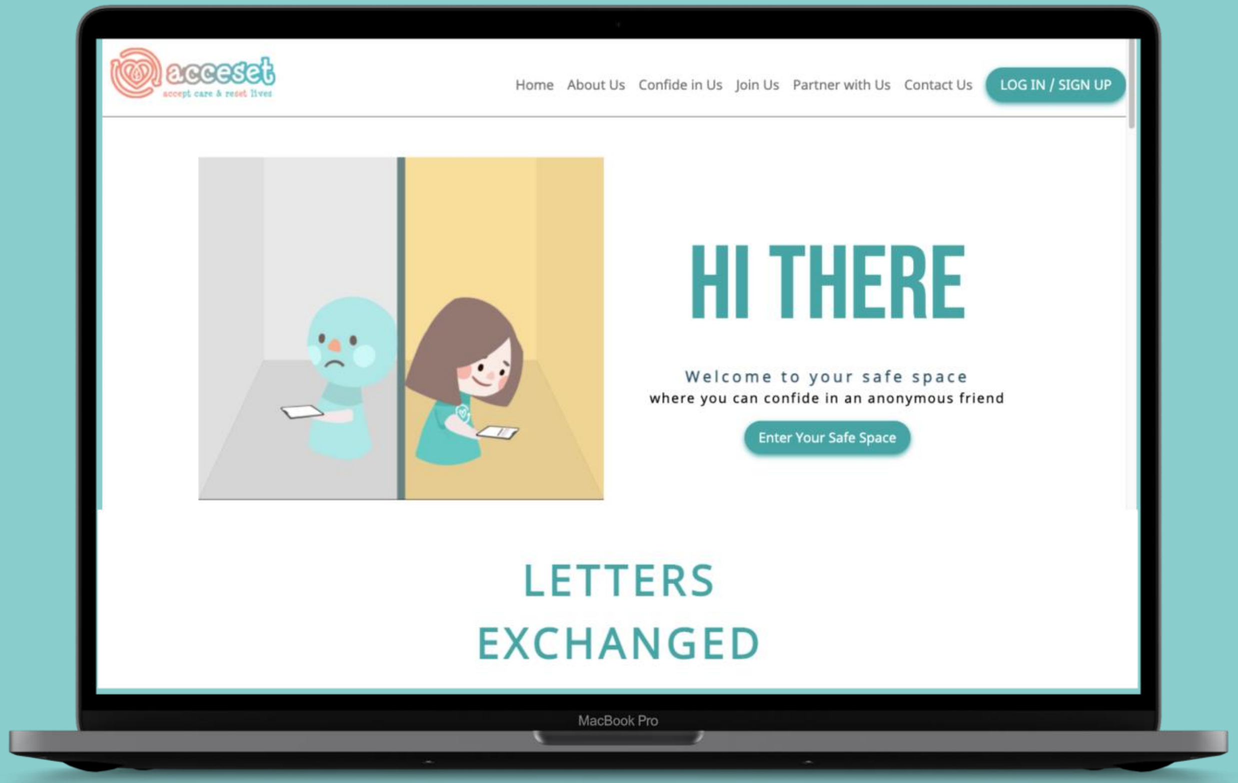

WHAT IS ACCESET?

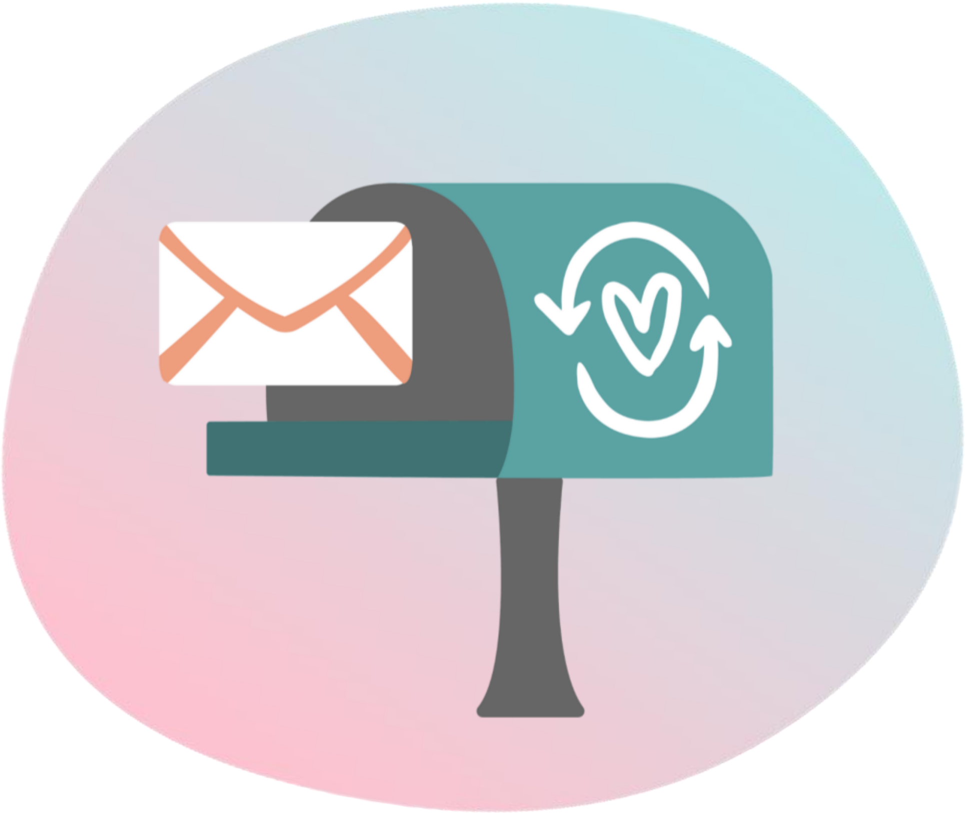

Anonymous Digital Letter Writing Platform

- Encourages students to share their challenges **anonymously** and **empathize** with their peers
- Students can exchange letters with **befrienders** trained in **Digital Peer Support Skills**

PRODUCT VIDEO

Please access the 90-sec video at this link: [https://nus.acceseet.com/video](https://nus.acceset.com/video)

acceset
accept care & reset lives

3 KEY ROLES

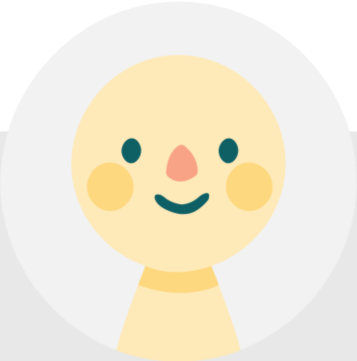

Seeker

A distressed and anonymous user who send and receive letters anonymously

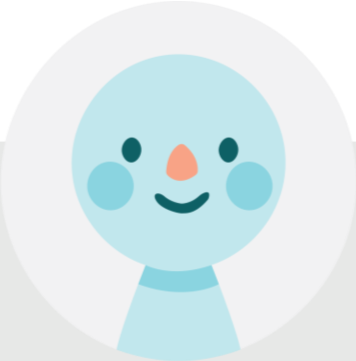

Befriender

A trained volunteer who replies letter from the seeker on an anonymous basis.

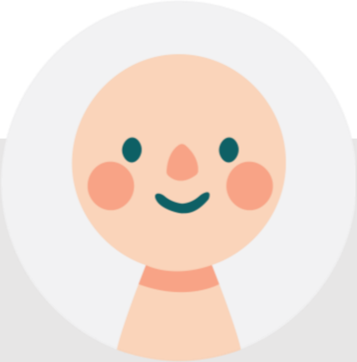

Moderator

A qualified mental health professional who reviews and vet befriender's reply

HOW ANONYMOUS LETTER EXCHANGE WORKS

01

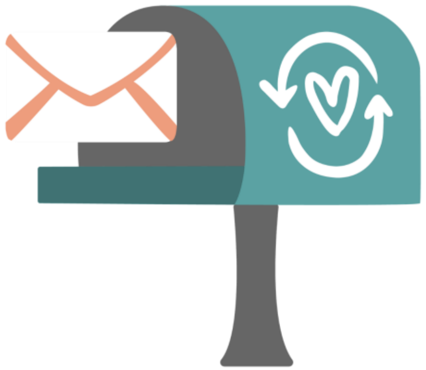

Seeker will first submit a letter anonymously

02

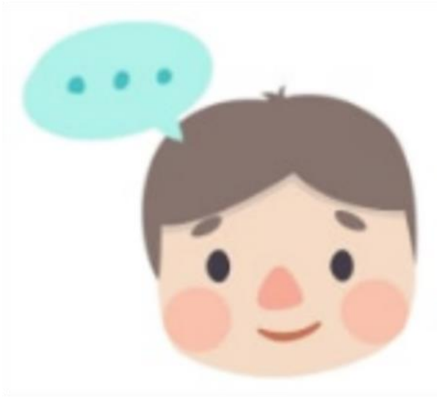

A trained Befriender will reply

03

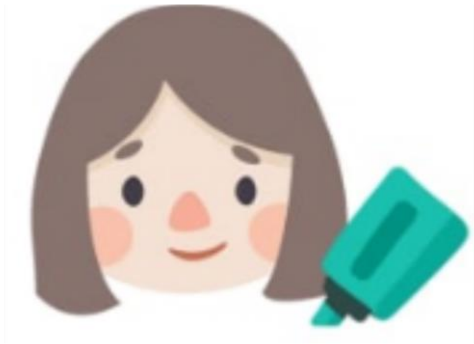

A Counsellor will moderate and vet the reply

04

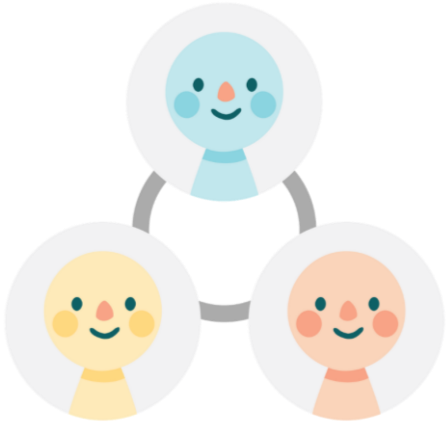

21 days countdown timer start once reply is sent out

WHY PARTNER US: DATA COLLECTED

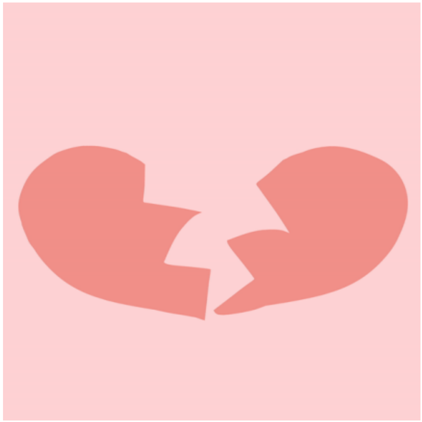

User Motivation

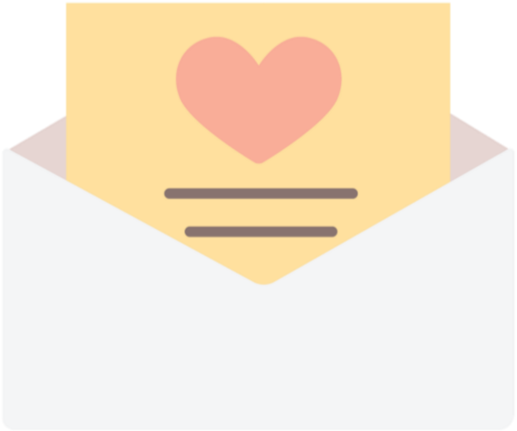

Letter Topics

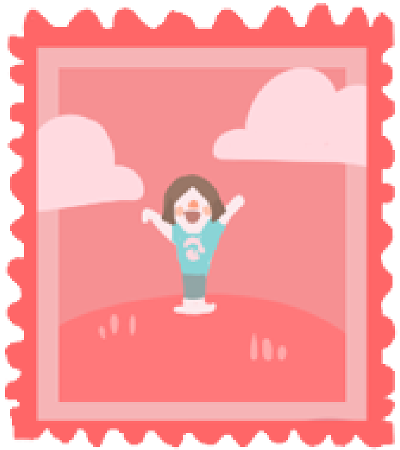

Emotion Sentiments

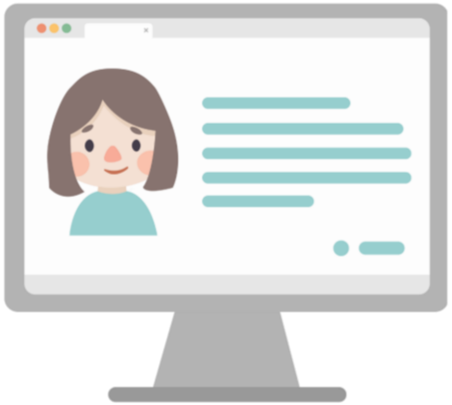

Unique User Demographics
