# Supplementary material for: A Digital Peer Support Platform to Translate Web-Based Peer Support for Emerging Adult Mental Well-being: Protocol for a Randomized Controlled Trial
Source: JMIR Res Protoc. 2022 Sep 20;11(9):e34602. doi: 10.2196/34602 (PMC9533208; doi:10.2196/34602)
Supplement: Multimedia Appendix 2 [file resprot_v11i9e34602_app2.pdf]

Multimedia Appendix 2. Components of mental well-being extracted from consultations with youths about their lived experiences.

Youth	Demographics		Psychological symptoms	Four Active Ingredients of Youth Mental Well-Being			
	Age	Gender		Mattering	Selfhood	Compassion	Mindfulness
#1	14-24	Male	Social anxiety	Being in your inner social circle is not the same as being significant	There needs to be reduced focused on the independent self but an increased focus on the interdependent self	Be kind to yourself, because fear and shame are a very big part of the experience<br><br>Feeling a sense of loneliness stemming from the fear and shame	The idea of having a 'toxic friend' inside my head that controls your actions<br><br>Learning how to mediate what that voice says and control the voice instead of the voice controlling you
#2	14-24	Male	General anxiety disorder (GAD) and short period of depression	A sense of mattering helps mitigate the loss of control, but does not help once in a panicked state<br><br>Advice is not important; validation of concerns is	A greater fear of the self than the fear of others	Know that a significant relationship is not always a healthy one and be ready to let it go	Mental imagery and grounding is very helpful in regaining a sense of control<br><br>Experience a lost of control in recognizing sources and triggers of anxiety
#3	14-24	Female	Depression	People with depression have an 'anxious-avoidant' attachment style<br><br>If we talk about mattering, what is significant is not	There is a very pessimistic outlook on life and a negative perception of the self that is part of the experience of depression	There is a sense of loneliness in having a mental health disorder because people can't really relate to your experience. Even if they do, depression	Understanding and accepting that episodes of depression were acts of purging pain

				what was said, but rather, who said it		makes you push them away because of the fear of vulnerability<br><br>It is easier to reach out to people with lived experiences as well because you don’t fear judgement	
#4	14-24	Female	GAD and depression	Depression or any mental illness adds to your sense of feeling unimportance<br><br>Mental illness soon became like a ‘bargaining chip’ because she learnt that when her psychiatrist called her parents to say she was having another episode, they would come	Self-harm also reduces the insignificance; it makes you feel like you have power to worsen the situation or to externalise the pain<br><br>Art therapy helped a lot. got better when she no longer let her mental state be defined by her achievement<br><br>A abusive relationship is the lesser of two evils - negative and hurtful attention is better than no attention and one is predisposed to choose negative over no attention because also a loss of sense of self/self-doubt/low self-esteem	Being aware that her depression was grounded on for a very young age because of the adverse childhood experience (ACE) of her mother leaving and father being very distant	Be purposeful in talking about what happened helps a lot with regaining control and staying in the present; you are intentional in deciding when it happens on your terms

#5	14-24	Female	Depression	Societal/parental expectations led to her depression and constant feeling of not being good enough	Slowly gained self-awareness to analyse her experiences of growing up and resulting depressive symptoms	Group therapy helped because the sense of feeling alone is diminished<br><br>Finds that solo travelling secures her a sense of freedom	She never felt like she was in the present moment. The episodes of depression was due to this, and she also attempted to be intentional of what she ate and how she is feeling
#6	14-24	Female	Depression	She had 'high functioning' depression as she always wanted to do everything because she felt like she had to earn her mum's attention<br><br>Fixation on awards and recognition because she felt like she mattered to others when she helped them		Her affection for life drives hope and optimism. It is her ability to be with people and express concern that relieve her depressed state	To get better, you need to accept where you are and slowly try to build a path to climb every mountain through means of coping mechanisms
Common patterns in the role of self-hood				• There is a greater fear of the self, more than the fear of others<br>• Feeling of low self-worth and having self-worth are tied to external achievements<br>• Self-awareness helps in identifying the triggers and sources of meltdowns			
Common patterns in the role of mattering				• A great sense of insignificance and needing to find means of being significant<br>• Feeling less alone or being assured that the journey is not done alone helps (whether it is through a shared experience or having someone to talk to). One can be seen to have many friends, but this does not matter if the people who seem to care about them are not the ones that matter, and if they do not fit in or do not feel they matter to the community that they are			

	a part of<br>• Feeling like they are not good enough will reinforce a sense of insignificance, even if they have social networks and friends (how selfhood relates to mattering).
Common patterns in the role of compassion	• Learning to be kind to themselves, particularly with their experiences of psychological symptoms<br>• Expressing concern and being supportive in their interactions and relations with others<br>• Helping people or doing things for them makes them feel useful, thereby increasing their feeling of significance and relieving their symptoms
Common patterns in the role of mindfulness	• Being aware of how one's feeling, thoughts and behaviors contributes to lived experiences help them manage the psychological symptoms<br>• Accepting one's psychological symptoms and lived experiences is key to coping<br>• Developing a sense of purpose by gaining clarity in personally meaningful aims, paths and values is integral to their well-being
