# Supplementary material for: A Digital Peer Support Platform to Translate Web-Based Peer Support for Emerging Adult Mental Well-being: Protocol for a Randomized Controlled Trial
Source: JMIR Res Protoc. 2022 Sep 20;11(9):e34602. doi: 10.2196/34602 (PMC9533208; doi:10.2196/34602)
Supplement: Multimedia Appendix 3 [file resprot_v11i9e34602_app3.pdf]

Multimedia Appendix 3. Questionnaire battery to assess the variables in the study

Type of Variables	Measures	Time-point	Informant/Respondent
Covariate(s)	**Letter content**<br>• number of letters<br>• length of letters<br>• Emotionality of letters, specifically the intensity of positive and negative emotions<br>• Quality of the content	T1-T4	Seekers' letters
	**Seekers' satisfaction**<br>• Client Satisfaction Questionnaire (CSQ-8)	T2	Seekers
	**Client Satisfaction** (for the befriender training program)<br>• Client Satisfaction Questionnaire (CSQ-8)	T4	Befrienders
	**Other factors**<br>• Participants' presenting conditions that may moderate effects of the interventions, such as gender and age.	T1	Seekers
Implementation outcomes	**Feasibility & Acceptability**<br>• initial and sustained engagement of seekers, befrienders and moderators (i.e., certified counsellor)<br>• use of technical features of the Acceset platform<br>• identification of participants with high risk for mental health conditions relating to depression and suicidality	T1-T4	Seekers, Befrienders, moderators
	**Acceset digital markers of psychological well-being**<br>• emotion sticker pack<br>• functional improvement<br>• motivations	T1-T4	Seekers' letters
Clinical outcomes	**Psychological well-being**<br>a. Anxiety (GAD-7)<br>b. Depression (PHQ-9)	T1-T4	Seekers, Befrienders

	**Four components of youth psychological well-being: Mattering, Self-hood, Compassion, Mindfulness**<br>• Before and after digital peer support training<br>• Coders to these 4 ingredients<br>• qualitative measure/data extracted from e-letter exchanges	T1-T4	
	1. **Perceived Social Support**<br>• Multidimensional Scale of Perceived Social Support (MSPSS)	T3-T4	Seekers

Note. T1 = time 1, at the start of the study; T2 = time 2 (3 weeks; after Acceset Intervention); T3 = time 3 (6 weeks); T4 = time 4 (9 weeks; end of study).
