# Supplementary material for: A Digital Peer Support Platform to Translate Web-Based Peer Support for Emerging Adult Mental Well-being: Protocol for a Randomized Controlled Trial
Source: JMIR Res Protoc. 2022 Sep 20;11(9):e34602. doi: 10.2196/34602 (PMC9533208; doi:10.2196/34602)
Supplement: Multimedia Appendix 4 [file resprot_v11i9e34602_app4.pdf]

Multimedia Appendix 4. Digital Peer Support Training Curriculum

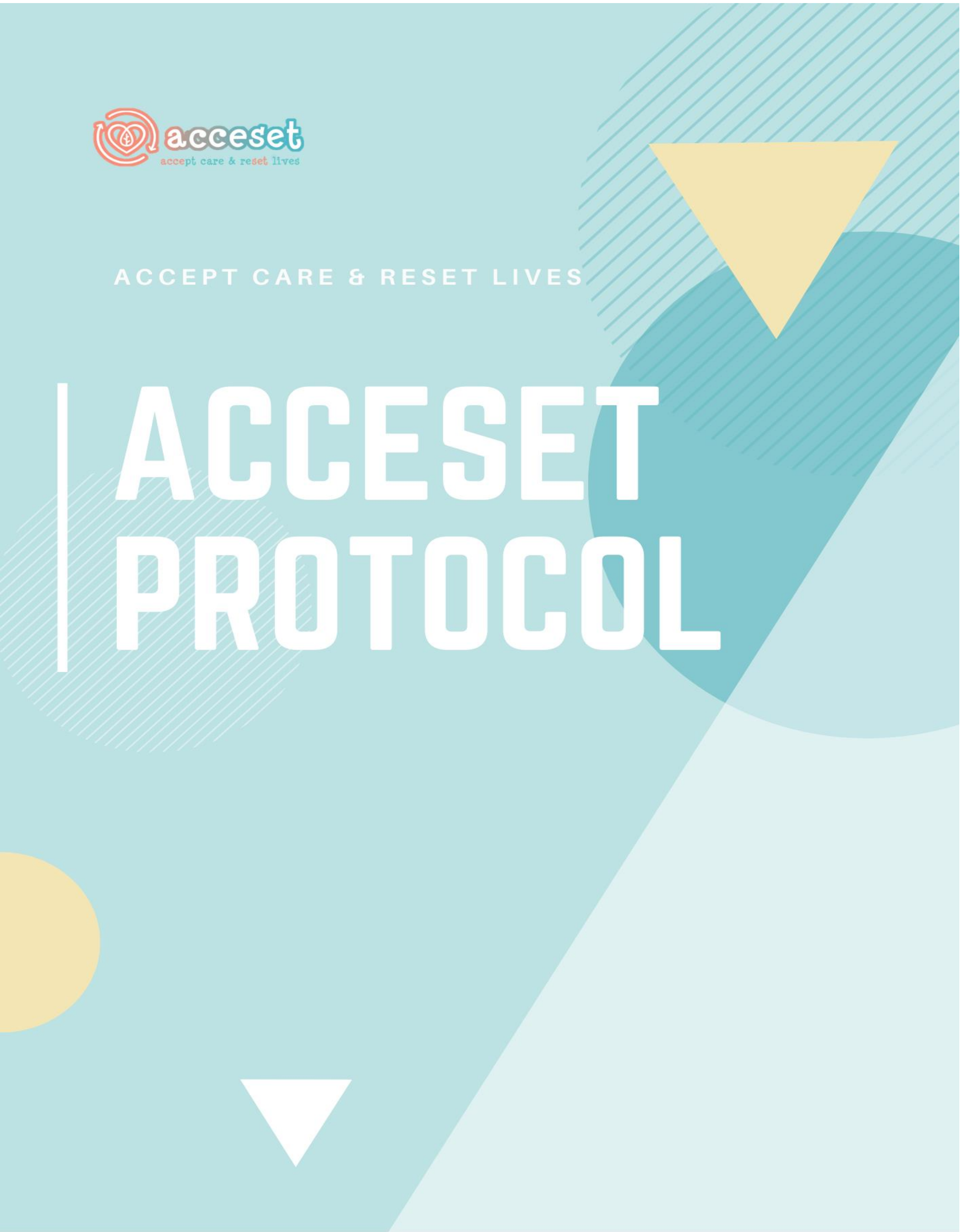

TABLE OF CONTENTS

Course description

- **A.** Learning objectives
- **B.** Learning outcomes

Procedure

- **A.** Procedure summary
- **B.** Program Breakdown

Appendix

Appendix A: Homework for Befrienders
Evaluation for Befriender homework

Appendix B: Acceset Sample Letters for Befrienders

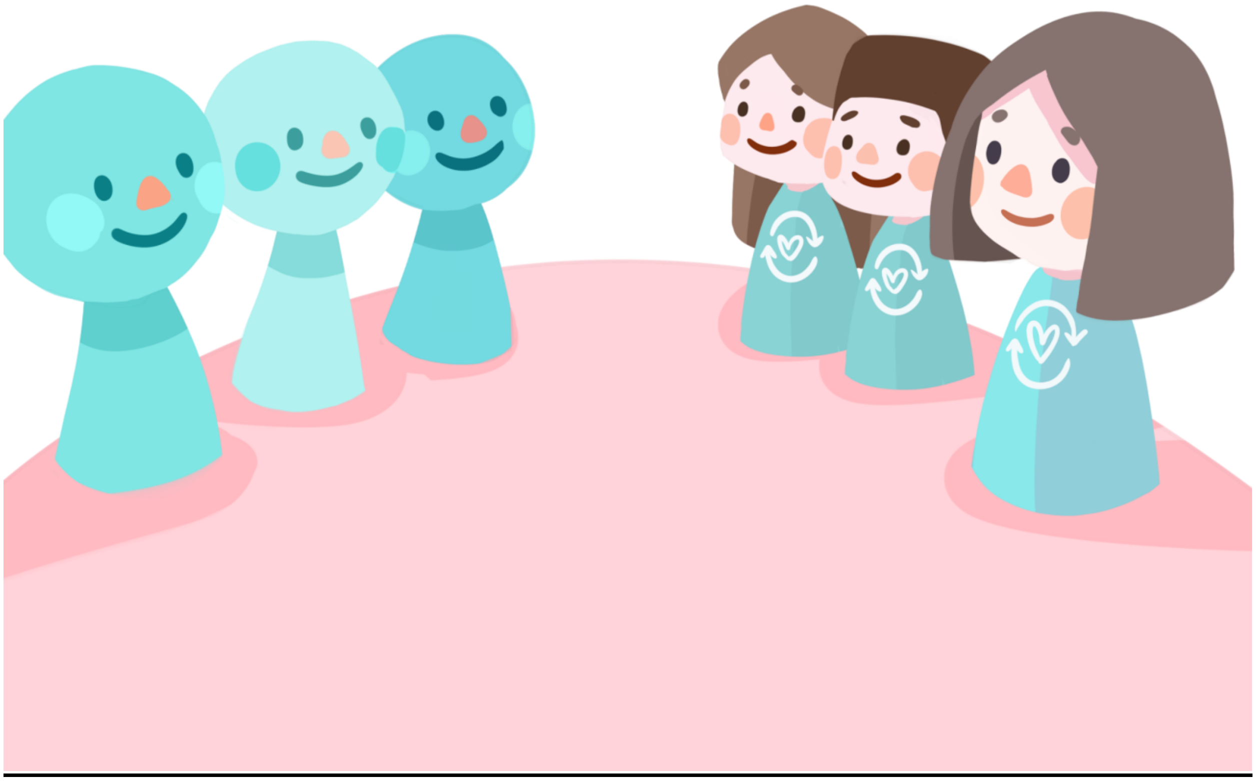

Course Description

This protocol presents Acceset's curriculum for digital peer support training. The purpose of the course is to equip individuals with the needed understanding and skills to help a friend in need in a precise and conducive manner that results in the friend feeling supported and empowered. The digital peer support training aims to develop the trainees' capacity to support others with skills built around how to enhance seeker's sense of mattering, addressing seeker's self-hood concerns, exploring compassion, and emphasizing personal growth. The concepts and practical skills of mattering, self-hood, compassion and personal growth will be explored and explained. The understanding and proficiency of these digital support skills will be sharpened through a simulation activity and homework. At the end of the training, trainees will walk away confident and ready to properly handle digital social situations where a friend is in need with a good outcome.

A. Learning objectives

- Enhance one's understanding of mattering, self-hood, compassion, and personal growth
    - Define mattering, self-hood, compassion and personal growth
    - Distinguish between mattering, self-hood, compassion and personal growth

- Enhance the capacity to support others with digital peer support skills
    - Be familiar with the process of mattering; cultivating self-hood; fostering compassion; and experiencing personal growth
    - Understand that mattering, self-hood, compassion and personal growth are interwoven and reinforce each other

- Apply digital peer support skills through simulation activity
    - Demonstrate the process of mattering through relating to the seeker
    - Demonstrate the process of enhancing compassion through psychological decentering
    - Demonstrate the process of enhancing personal growth through empowerment and helpful thinking
    - Demonstrate the process of cultivating self-hood through problem-solving

B. Learning outcomes

- Enhance seeker's sense of mattering
- Addressing seeker's self-hood concerns
- Exploring compassion
- Emphasizing personal growth

Enhance seeker's sense of mattering	
Understand the 3 components of mattering	1. Quality Attention<br>2. Developing Importance<br>3. Recognizing Reliance

Know how to relate to the seeker	1. Appreciate and make the seeker feel important to build up the interpersonal self of the seeker<br>2. Build trust with the seeker<br>3. Ask follow-up question

Addressing seeker's self-hood concerns	
Understand 3 aspects of self-hood	1. Self-knowledge (social actor)<br>2. Interpersonal self (motivated agent)<br>3. Self as agent (autobiographical author)
Develop self-hood through problem-solving	1. Brainstorm the different options and choices to cope with the immediate stressor<br>2. Implement the best approach and review outcomes<br>3. Appraise the pro and con of different coping methods to manage the stressors<br>4. Identify an immediate stressor or root cause<br>5. Strengthening selfhood through mattering

Exploring compassion	
Understand the 3 orientations of compassion	1. Having compassion for others<br>2. Being the object of compassion<br>3. Having compassion for oneself
Understand the 4 stages of compassion	1. The awareness of suffering<br>2. An affective concern for others<br>3. A wish to relieve that suffering<br>4. A readiness to relieve that suffering
Enhancing compassion through psychological decentering	1. Observation of cognitive thought<br>2. Depersonalize or acceptance<br>3. Relate to self-knowledge and strength

Emphasizing personal growth	
Understanding mindfulness as personal growth	1. Mindfulness is a disposition to the present moment
Learn the benefits of mindfulness	1. Improve emotion regulation<br>2. Improve anxiety and depression<br>3. Boost positive mood
Learn how to enhance personal growth	1. Empowerment and helpful thinking<br>2. Practicing mindfulness

Procedure

A. Procedure summary

Befrienders will receive 4 hours training in digital peer support skills, which consist of 1 hour of instructional content that enhances the befriender's knowledge of mattering, self-hood, compassion and personal growth, and 3 hours of workshop training that enhances the befriender's capacity to support others. They will learn therapeutic approaches such as problem-solving, emotion regulation and grounding skills. They will be required to complete a simulation activity that tests the application of these skills to mock letters that are adapted from actual cases.

This training will be based on the digital peer support skills so that they can positively relate to seekers. Their focus will be meeting the seekers psychological needs, rather than providing advice and diagnosis of mental health problems. All the participant's classroom assignments will be reviewed and given feedback. Participants who complete the training will receive a training certificate and will be invited to take on live cases and be part of the study.

The training program on understanding mattering, self-hood, compassion, and personal growth and how to provide digital support is developed by Acceset together with 6 other charities, namely Boy's Town, Campus PSY, Singapore Children's Society, Singapore Association of Mental Health, Caregiver's Alliance and Limitless. The training will be provided by Acceset, specifically, conducted by Matt, who is the curriculum developer. The befrienders will learn the digital support skills (i.e. how to positively relate to a peer). Specifically, the type of text-based support they provide to seekers revolve around restoring a sense of significance, addressing the seeker's psychological needs as well as discussing the components of positive well-being. Below, we illustrate the key skills of digital peer support, namely mattering seekers, how to address the seeker's psychological needs and how to facilitate discussion of the components of well-being.

B. Program Breakdown

PROGRAM BREAKDOWN
**Part 1: Understanding Mattering, Self-Hood, Compassion and Personal Growth (60mins)**<br>• ***Introducing Mattering***<br>○ Definition of mattering<br>○ The process of mattering<br>• ***Introducing Self-Hood***<br>○ Definition of self-hood<br>○ How to develop a strong sense of self<br>• ***Introducing Compassion***<br>○ Definition of Compassion<br>○ The process of compassion<br>• ***Introducing Personal Growth***<br>○ Definition of Personal Growth - mindfulness<br>○ Benefits of mindfulness

Part 2: Learn and Apply Mattering, Self-Hood, Compassion and Personal Growth (45 mins)

- ***Mattering***
    - Mattering - relating to the seeker via appreciation and importance to build up the interpersonal self
    - Mattering – Build trust, relate with your own experience, ask follow-up questions.
- ***Self-hood***
    - Self-hood - Self-knowledge, Interpersonal Self, Self as Agent
    - Cultivating selfhood through collaboration and peer support - cultivate problem-solving skills to build up self as self-hood
    - Problem-solving steps – Identify goal, brainstorm different means to get there, appraise best approach, implement and review
    - Examples of application
- ***Compassion***
    - Compassion through psychological decentering
    - Psychological Decentering – separate identity and external experience
    - How to decenter – Validate ideas, list out what can/cannot be controlled, affirm strengths
- ***Personal growth***
    - Empowerment – focus on non-negative information
    - Reduce self-criticism through reassurance via mattering and psychological decentering.
    - Resilience – Problem-solving, People, Passion

Part 3: Homework

After training, befrienders are required to submit a response to one of the sample letters of their choice to the admin portal. The trainer will then vet their responses and evaluate whether the befrienders have applied the framework.

Appendix A

Homework for Befrienders

After training, befrienders are required to submit a response to one of the sample letters of their choice to the admin portal. The trainer will then vet their responses and evaluate whether the befrienders have applied the framework.

Evaluation for Befriender homework

Participants are required to submit one written assignment for assessment. Responses will be awarded between 1 and 5 (1 being poor and 5 being excellent) on the following criteria:

1. Capacity for compassion: to empathize – demonstrates evidence of attending to the feelings and issues narrated of the seeker by means of distilling key points raised by the seeker and paraphrasing them.
2. Capacity to enhance personal growth: develop and communicate positive and affirming thoughts – demonstrates evidence of helping the seeker to shift their attention from an information or stimuli that is perceived to be negative to non-negative/neutral/positive stimuli/information. For example, while I empathize with your worries about being a burden and how you find it hard to be truthful ("negative stimuli"), I want to also affirm your strength and courage in sharing with me how you truly feel which I think is a very brave thing to do ("non-negative stimuli").
3. Capacity for mattering: ability to develop a sense of significance in seeker – demonstrates evidence of being interested in the seeker's issues by asking follow-up questions to content raised by the seeker or identifying the strengths that the seeker has displayed or facilitating discussions that helps the seeker make sense of their identity.
4. Capacity to cultivate self-hood: facilitate problem-solving thinking in the seeker – Demonstrates an ability to prioritize key issues that troubles the seeker, identify goals and objectives that enables the seeker to brainstorm and think through ways to overcome problems.

Participants are required to obtain a score of 12 or more to qualify to be a befriender.

Befriender Response Evaluation

Evaluation Criteria:

Explore compassion by validating the negative experiences (feelings and thoughts) of the seeker

Rating given	Evaluation criteria: Ability to be compassionate
Poor	Lacks ability to accurately name the troubling emotion that the seeker is experiencing, instead misinterpreting the emotion experienced by the seeker. Alternatively, may make dismissive comments about seeker's feelings that cause the seeker to feel invalidated. May also switch to irrelevant topics or talk about how to problem-solve without first validating the seeker's feelings.
Fair	Lacks ability to accurately name the troubling emotion that the seeker is experiencing, instead misinterpreting the emotion experienced by the seeker.
Good	Ability to accurately name the troubling emotion that the seeker is experiencing, only in situations when it is explicitly expressed in message content by the

	seeker. Also capable of paraphrasing and reflecting back what the seeker has said, to check that message has been accurately understood.
Very Good	Ability to accurately name the troubling emotion that the seeker is experiencing, only in situations when it is explicitly expressed in message content by the seeker. Also capable of paraphrasing and reflecting back what the seeker has said, to check that message has been accurately understood.<br>Furthermore, conveys the idea (with reasons) that the seeker's feelings and behaviour are understandable given the seeker's past experiences or present situation.
Excellent	Ability to accurately name the troubling emotion that the seeker is experiencing, even when it is implicit (i.e., has to be inferred from the message content). Also capable of paraphrasing and reflecting back what the seeker has said, to check that message has been accurately understood.<br>Furthermore, conveys the idea (with reasons) that the seeker's feelings and behaviour are understandable given the seeker's past experiences or present situation.

Demonstrate mattering by distinguishing the seeker's positive qualities and identity from their negative experiences

Rating given	Evaluation criteria: Cultivating a sense of mattering
Poor	May neglect to validate the seeker's personal qualities or strength; give trite compliments that are inappropriate for or irrelevant to seeker and the situation the seeker is in; or blame the seeker for the negative experience that he/she is in.
Fair	May neglect to validate the seeker's personal qualities or strength.
Good	Ability to give attention to and genuinely validate the seeker's personal qualities and strengths that are appropriate for the seeker's problem context.
Very Good	Ability to give attention to and genuinely validate the seeker's personal qualities and strengths, after mild consideration of the existing information about the seeker and the situation the seeker is in. The chosen personal quality or strength that is being validated is somewhat appropriate for the seeker's demographic (e.g. age, gender, SES) and problem context.
Excellent	Ability to give attention to and genuinely validate the seeker's personal qualities and strengths, after thoughtful consideration of the existing information about the seeker and the situation the seeker is in. The chosen personal quality or strength that is being validated is entirely appropriate for the seeker's demographic (e.g. age, gender, SES) and problem context. Also communicates idea that the negative circumstance does not define the seeker.

Enhance personal growth by focusing seeker's attention on neutral or positive information from the issue

Rating given	Evaluation criteria: Ability to enhance personal growth
Poor	Dwells on the negative stimulus that is bothering the seeker, by extensively talking about how bad the situation is. Encourages the seeker to continuously share more negative details about the situation or vent excessively, potentially amplifying seeker's negative emotions.
Fair	Encourages seeker to share more negative details about the situation or vent to a moderate extent, but balances out with more neutral or positive topics.

Good	Encourages the seeker to share briefly about the negative details about the situation or vent, but makes attempts to neutralise the negative stimuli.
Very Good	Encourages the seeker to share briefly about the negative details about the situation or vent, but makes attempts to neutralise the negative stimuli, and also re-frames the negative stimuli such that it reflects more positively.
Excellent	Encourages the seeker to share briefly about the negative details about the situation or vent, and validates the negative emotions; but makes attempts to neutralise the negative stimuli, and also re-frames the negative stimuli such that it reflects more positively. May also ask the seeker questions to encourage him/her to reflect on possible alternative ways to look at the situation.

Cultivating self-hood by brainstorming with the seeker different ways to solve the problem

Rating given	Evaluation criteria: ability to cultivate self-hood
Poor	Lectures the seeker for using a poor problem solving approach and tells the seeker what to do based on one's own personal experience.
Fair	Lists out possible solutions that the seeker can try, or asks the seeker if they have tried those suggested solutions as of yet.
Good	Asks questions to prompt the seeker to reflect on goals and whether the current problem solving approach used is helpful in meeting the seeker's goals. Provides suggestions on what the seeker can do to meet his/her goals.
Very Good	Asks questions to prompt the seeker to reflect on goals and whether the current problem solving approach used is helpful in meeting the seeker's goals. Does a mix of asking questions to guide seeker in thinking what he/she can do to meet his/her goals, and providing helpful suggestions in a tentative manner.
Excellent	Asks questions to prompt the seeker to reflect on goals and whether the current problem solving approach used is helpful in meeting the seeker's goals. Asks questions to guide seeker in thinking what he/she can do to meet his/her goals and in planning concrete steps to do so, and providing helpful suggestions.. Also engages the seeker in reflecting on what is still going well in the situation and how to increase more instances of it.

Appendix B

Acceset Sample Letters for Befrienders

Letter 1
Topic: Family

Hi, I feel really unhappy with my family. My mum will bring home problems from work. Once she was telling me about how her colleagues told her off and scolded a lot of foul words. Why does she have to burden me with her adult issues and bring so much angst home? My father gambles every afternoon and so I just play com games or watch TV when I'm home from school because I'm all alone. I don't get any outings or fun stuff even on weekends because my parents aren't around. I try asking my friends to go out with me but they always have family stuff… I feel this shows that my classmates' parents worry and plan for their children but my parents do not care about me.

My results are quite bad also, and everyone feels like I'm useless. Is the way our society functions, where people are quick to judge? I've always experienced poor concentration since young, and I have met people who tried to take advantage of my condition and even thinks I'm an idiot. Once I tried talking to my teacher, but she wasn't helpful she just said like "Just try harder". But how to do that when I'm already trying my best???

Letter 2
Topic: Relationship

I've been in this relationship for almost 6 months. My girlfriend has really low self esteem and according to her, she has anxiety and gets panic attacks. She's really really possessive and hates it when any girl pays attention to me. I supposedly can't talk to the girls in my class or hang out with a group of friends which has girls in too. It's really difficult that I've got no space to socialize like I always do, and sometimes it's impossible because I need to work with girls for CCA or projects. Recently we had a fight which led us to almost breaking up, but I think I love her so much that I tried to save it. It started when I walked to the bus stop with a girl from school and her friend saw it and told her. My girlfriend got so jealous that she told me to stop talking to the girl and always text her when I'm leaving school. It's not that i don't love her, but I really think it's harmless to walk to the bus stop with a schoolmate, no?? She gave me paragraphs of texts about how insensitive I am and how I ignore her insecurity and her feelings.

Whenever I try to tell her how I feel, she always brings up her ~~"trauma"~~ (negative experiences) and thinks that what she's doing is right. We fight at least once a week and I hate it so much. But then she'll apologise and say that she'll improve or that she couldn't help it. What else can I do? I have to just keep forgiving but I see no point in trying to tell her how I truly feel. I wish I had the courage to pull myself together and leave but i've got no heart to, I still love her despite whatever has happened.
